# Influence of the Length of the Alanine Spacer on the Acidic–Basic Properties of the Ac–Lys–(Ala)_*n*_–Lys–NH_2_ Peptides (*n* = 0, 1, 2, …, 5)

**DOI:** 10.1007/s10953-012-9903-7

**Published:** 2012-10-13

**Authors:** Joanna Makowska, Adam Liwo, Lech Chmurzyński, Harold A. Scheraga

**Affiliations:** 1 Faculty of Chemistry, University of Gdańsk, Sobieskiego 18, 80-952 Gdańsk, Poland; 2 Baker Laboratory of Chemistry and Chemical Biology, Cornell University, Ithaca, NY 14853-1301 USA

**Keywords:** Alanine-based peptides, Potentiometric titration, Acid–base equilibria

## Abstract

By using the potentiometric titration method, we have determined the p*K*_a_ values of the two terminal lysine groups in six alanine-based peptides differing in the length of the alanine chain: Ac–Lys–Lys–NH_2_ (KK), Ac–Lys–Ala–Lys–NH_2_ (KAK), Ac–Lys–Ala–Ala–Lys–NH_2_ (KAK2), Ac–Lys–Ala–Ala–Ala–Lys–NH_2_ (KAK3), Ac–Lys–Ala–Ala–Ala–Ala–Lys–NH_2_ (KAK4), and Ac–Lys–Ala–Ala–Ala–Ala–Ala–Lys–NH_2_ (KAK5) in aqueous solution. For each compound, the model of two stepwise acid–base equilibria was fitted to the potentiometric-titration data. As expected, the p*K*_a_ values of the lysine groups increase with increasing length of the alanine spacer, which means that the influence of the electrostatic field between one charged group on the other decreases with increasing length of the alanine spacer. However, for KAK3, the p*K*_a1_ value (8.20) is unusually small and p*K*_a2_ (11.41) is remarkably greater than p*K*_a1_, suggesting that the two groups are close to each other and, in turn, that a chain-reversal conformation is present for this peptide. Starting with KAK3, the differences between p*K*_a1_ and p*K*_a2_ decrease; however, for the longest peptide (KAK5), the values of p*K*_a1_ and p*K*_a2_ still differ by about 1 unit, i.e., by more than the value of log_10_ (4) = 0.60 that is a limiting value for the p*K*_a_ difference of dicarboxylic acids with increasing methylene-spacer length. Consequently, some interactions between the two charged groups are present and, in turn, a bent shape occurs even for the longest of the peptides studied.

## Introduction

The protonation state of ionizable side-chain groups of peptides and proteins is of great importance for understanding biomolecular structure and function, and therefore much effort has been devoted to understanding the relation between p*K*_a_ and the structures of biomolecules [1–6]. The protonation state of a titratable group is determined by the solvent pH and the p*K*_a_ of that group. The p*K*_a_ of a given group is in turn influenced by its electrostatic environment, which is determined by the protein’s conformation.

Alanine-based peptides, with the general formula XX–(A)_*n*_–YY, where X and Y denote an amino acid residue with an ionizable side chain, have received much attention in recent years, because they are water soluble and can model the conformation of the oligoalanine chain as a function of chain length [7]. Since alanine is a small and neutral amino acid residue, such peptides offer an opportunity for investigating the mutual influence of ionizable groups on each other and on their conformation. In this respect, the peptide Ac–XX–(A)_7_–OO–NH_2_ (where X denotes diamino-butyric acid, A denotes alanine, and O denotes ornithine), which is referred to as XAO, has received considerable attention [8, 9]. In XAO, the ionizable side-chain groups of all four X and O groups are –NH_3_^+^.

We investigated the structure of this peptide [10, 11] by CD and NMR spectroscopy and concluded that it has a bent shape, and although the polyproline II structure appears for individual residues, it is not characteristic of the shape of the entire peptide. Potentiometric titrations [10, 11] yielded values of p*K*_a1_ = 3.46, p*K*_a2_ = 4.67, p*K*_a1_ = 6.35, p*K*_a2_ = 6.91, with p*K*_a1_ being abnormally low, which provides evidence for the proximity of the like-charged groups. The difference between the p*K*_a*i*_ values should decrease when the spacer becomes longer, though not to zero because of a statistical factor equal to 4 for *K*_1_/*K*_2_ of dicarboxylic acids with increasing methylene-spacer length [12].

In our recent work [7, 13], in order to determine the influence of the kind of ionizable groups flanking the oligoalanine sequence on acidic–basic and conformational properties, we investigated the following three alanine-based peptides: Ac–KK–(A)_7_–KK–NH_2_ (KAK), Ac–OO–(A)_7_–DD–NH_2_ (OAD), and Ac–KK–(A)_7_–EE–NH_2_ (KAE), where A, K, O, D, and E, denote alanine, lysine, ornithine, aspartic acid, and glutamic acid residues, respectively. For these peptides, we determined both p*K*_a_ values by potentiometric titration [7] with structural conformation using CD and NMR spectroscopy [13]. We demonstrated [13] that the shape of these peptides seems to depend on the size of the charged side chains at the ends, and that the bent shape of the alanine sequence enables screening of the nonpolar heptaalanine core from the solvent by the charged side chains, provided that the side chains bearing charged groups are sufficiently short. The results of our studies mentioned above [7, 10, 11, 13] enabled us to propose a hypothesis that the bent shape is characteristic of oligopeptides flanked with like- or oppositely-charged groups, provided that the length of the spacer (including the segments of the main chain and the side chains) between the charged groups is large enough to form a bent structure and small enough to overcome the ring-closure entropy. The peptides acquire a bent shape probably because the charged side-chain groups wrap around the non-polar oligoalanine–alanine sequence, thus shielding the non-polar residues from the solvent.

In the present study we investigated a series of water-soluble alanine-based peptides with the sequence Ac–K–(A)_*n*_–K–NH_2_, where *n* varies from 0 to 5, by potentiometric titration. The presence of only one basic residue at each end of the sequence enabled us to make a clearer assessment as to the influence of the length of the alanine spacer on the acidic–basic properties of these bi-cationic acids compared to the series with two different groups at each end, in which case the second p*K*_a_ can correspond either to deprotonation of the second basic group at the already singly-deprotonated end or to deprotonation of a basic group the opposite end.

## Methods

### Peptide Synthesis

The peptides Ac–KAAAK–NH_2_ [KAK3], Ac–KAAAAK–NH_2_ [KAK4], and Ac–KAAAAAK–NH_2_ [KAK5] (where K and A denote lysine and alanine, respectively), were synthesized by standard solid-phase Fmoc-amino acid chemistry with a Millipore synthesizer. Tentagel R RAM resins (1 g, capacity 0.19 mmol g^−1^) were treated with piperidine (20 %) in dimethylformamide (DMF), and all amino acids were coupled using the DIPCI/HOBt (*N,N′*-diizopropylokarbodiimid/1-hydroksybenzotriazol) methodology. The coupling reaction time was 2 h. Piperidine (20 %) in DMF was used to remove the Fmoc group at all steps. After deprotection of the last Fmoc N-terminal group, each of the resins was washed with methanol and dried *in vacuo*. Then the resins were treated with 1 mol L^−1^ 1-acetylimidazole in DMF at room temperature for 2 h to attach the acetyl group to the N-terminal part of the peptide (after a complete polypeptide chain was assembled). In the final step, the resins were treated with a TFA/water/phenol/triisopropylsilane (8.8/0.5/0.5/0.2) mixture (10 mL·g^−1^ of resin) at room temperature for 2 h to remove the peptide from the resin. Cleavage of the peptide from the resin under these conditions leaves a carboxyamide group at the *C*-terminus [14].

Each of the resins was separated from the mother liquid; the excess of solvent was then evaporated to a volume of 2 mL, and the residue was precipitated with diethyl ether. The crude peptides were purified by reverse-phase HPLC using a Supelcosil™ SPLC-ABZ C_18_ semi-preparative column (10 × 250 mm, 5 μm) with 4 mL·min^−1^ elution and 120 min with an isocratic mixture of 0.5 % CH_3_CN in TFA to adjust the pH to approximately 2.0. To identify the fractions containing the pure peptides, HPLC was run first with a small amount of the crude peptide and the absorbance at 222 nm was measured for each fraction. The time windows for the retention of purified peptides were determined as those corresponding to the large peak in the plot of absorbance versus time. Subsequently, a semi-preparative HPLC run was carried out and the fractions containing the pure peptide were collected and lyophilized. The purity of each peptide was confirmed by analytical HPLC and MALDI-TOF analysis. The purities of these peptides are: 99.82 % (KAK3), 99.4 % (KAK4), and 99.85 % (KAK5).

Peptides KK, KAK, KAK2 were also synthesized by the solid-phase peptide synthesis method but with a Thuramed TETRAS peptide synthesizer. The purity of each peptide, determined by analytical HPLC and MALDI-TOF analysis, are 96.2 % (KK), 97.43 % (KAK), and 94.57 % (KAK2).

### Potentiometric Titration

All measurements were carried out at *T* = 298.1 ± 0.1 K in an e.m.f. cell containing a microelectrode. The potentiometric microtitration unit was equipped with a computer-aided microtitrator system and a millivoltmeter with a resolution of 0.01 mV. The titrant was added drop wise using a 2.5 cm^3^ HAMILTON syringe with an accuracy of 0.001 cm^3^. Three independent titrations were carried out for each of the peptides studied. For each titration, a stock solution of the peptide was prepared at a concentration of approximately 0.001 mol·L^−1^, and 3 cm^3^ of this solution was added to the titration vessel. The solution of the peptide in water was titrated with 0.018 mol·L^−1^ NaOH in water. The p*K*_a_ value for water was not considered an adjustable parameter, a value of 14 being assumed. The starting pH values of the titrated solution of peptides are as follows: 4.4 (KK), 4.74 (KAK), 3.54 (KAK2), 2.70 (KAK3), 2.64 (KAK4), and 2.77 (KAK5). The initial ionic strength of the titrated solution of peptides was 0.003 mol·L^−1^; after complete titration by NaOH the ionic strength decreased to 0.002 mol·L^−1^ (because a completely charged peptide bears a +2 net charge, which is replaced by two singly-charged sodium cations after complete titration). The measurements were performed with at rate of 1 step per 2 min. For each titration point, the e.m.f. was recorded only after allowing sufficient time for electrode equilibration.

**Fig. 1:**
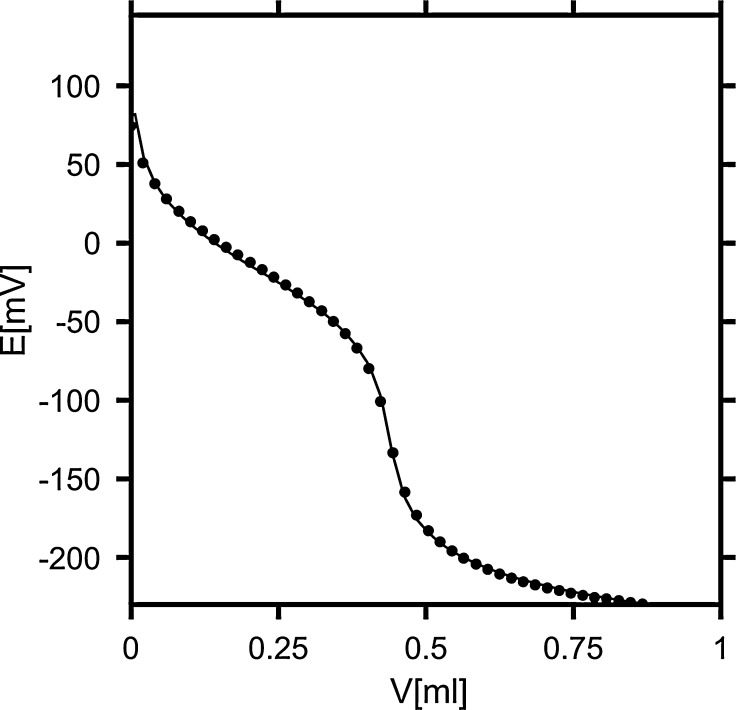
Calibration curve (experimental titration points for NaH_2_PO_4_ in water). Experimental data are shown as *points* and the curve corresponding to the best-fitting equilibria model is given by the *solid line*. The standard deviation in the e.m.f. is 1.34 mV

Before carrying out a series of measurements, the electrode was calibrated as follows. 3 cm^3^ of 0.001 mol·L^−1^ NaH_2_PO_4_ in water was titrated with 0.01 mol·L^−1^ aqueous solution of sodium hydroxide (NaOH); three independent titrations were carried out for reliability and better accuracy. The electrode characteristics were fitted to the titration curves from all three independent titrations simultaneously, using the program STOICHIO [15, 16]. The consecutive p*K*_a_ values of phosphoric acid were taken from the literature as p*K*_a1_ = 2.3, p*K*_a2_ = 7.2, and p*K*_a3_ = 12.1, respectively. The fitted values of *E*° and *S* in the equation for electrode characteristics (Eq. 1) were 394.8 (1.3) mV and 57.28 (0.13) mV, respectively [the numbers in parentheses are the corresponding standard deviations (SD)]. The SD of the electromotive force and the titrant volume were 1.3 mV and 0.001 cm^3^, respectively. The estimated error in pH is 0.02. This value is in agreement with the SD of the e.m.f. obtained after fitting, 1–2 mV, depending on the peptide. The fit of the calculated titration curve to a sample experimental titration curve is shown in Fig. 1.

1 $$ E = E^\circ - S\,{\text{pH}} $$

where *E* is the electromotive force and the pH is calculated at each point from the total concentration of the titrand and the titrant, volumes of the titrand and the titrant, the p*K*_a_s, and p*K*_w_.

### Fitting Models of Chemical Equilibria to the Titration Curves

**Scheme 1:**
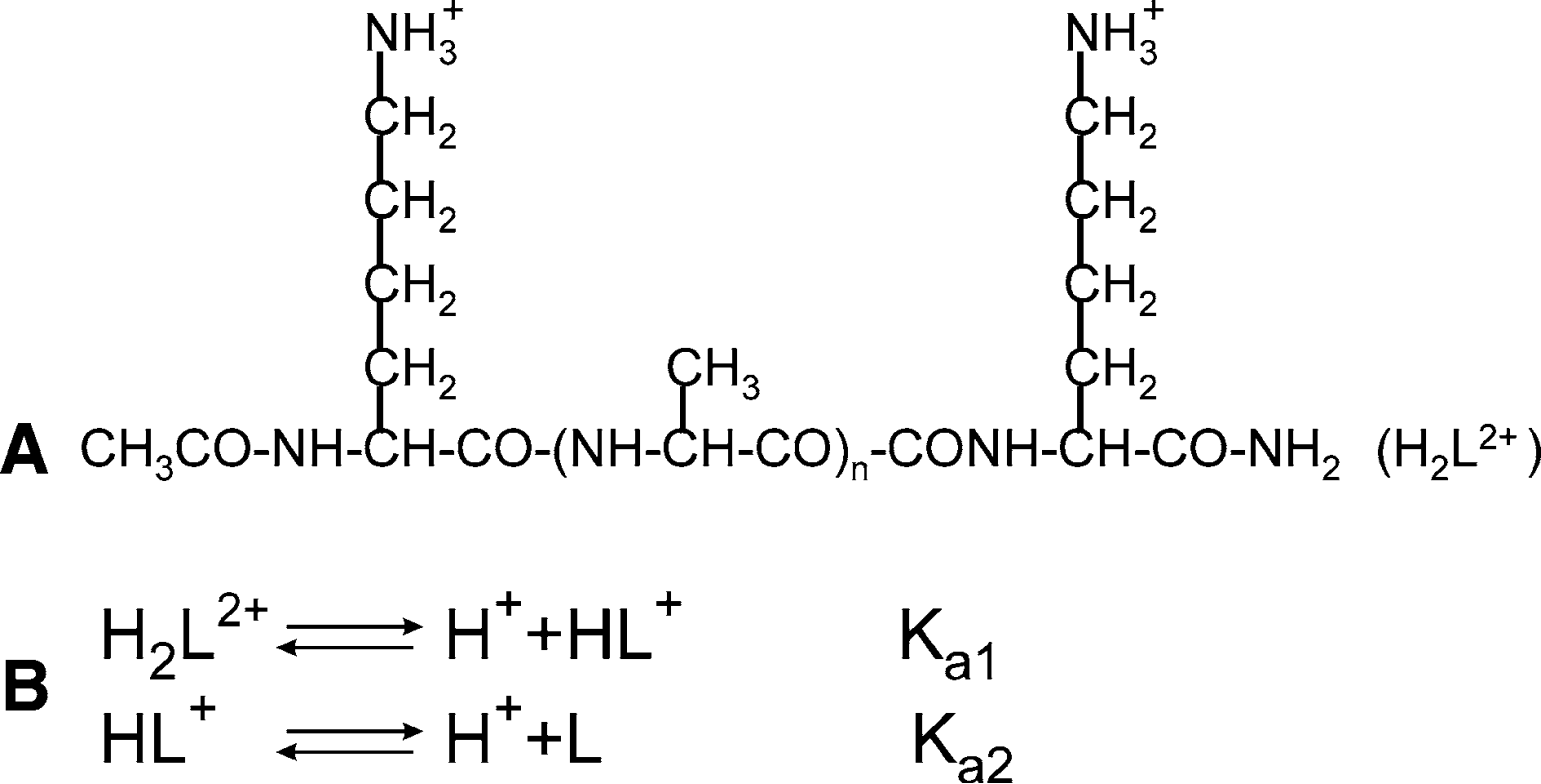
The structure of the peptides studied (**a**) and the acid–base equilibria involved in system (**b**)

The model for the acid–base equilibria was fitted to the resulting titration curves with the program STOICHIO [15, 16] which is based on a nonlinear confluence analysis. This program can treat any model of chemical equilibria, and takes into account all possible sources of experimental error (the error in e.m.f., titrant and titrated solution volume, reagent impurities, etc.; see [14, 15] for details of the determination of the p*K*_a_s). The SD of the titrant volume was assumed to be σ_*V*_ = 0.001 cm^3^ and σ_*E*_ = 2.0 mV. The first of these values is the accuracy of the microsyringe used to add the titrant to the titrated solution, while σ_E_ is much larger than the resolution of the potentiometer given as 0.01 mV in Sect. 2.2, to take into account possible electrode instability and other possible errors involved in measuring the e.m.f. as was done in our previous studies [7, 13]. Because all of the peptides studied were terminally-blocked by the acetyl group and by the amide group at the *N*- and at the *C*-termini, respectively, only the Σ-NH_3_^+^ groups of the lysine side chains could take part in acid–base equilibria. Consequently, all peptides under study were treated as bifunctional acids, and the 2 p*K*_a_ values were considered as quantities to be determined by the STOICHIO program. The general structure of the peptides studied and the acid–base equilibria scheme are summarized in Scheme 1.

## Results and Discussion

**Fig. 2:**
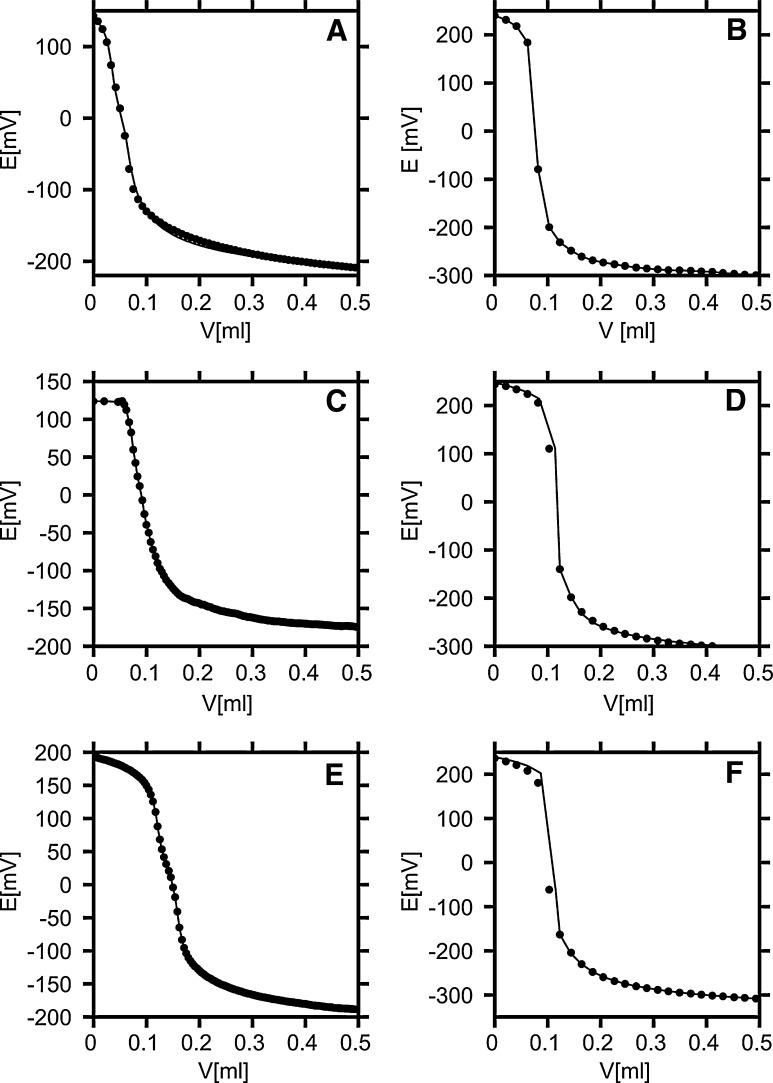
Experimental titration curves of KK (**a**), KAK (**b**), KAK2 (**c**), KAK3 (**d**), KAK4 (**e**), KAK5 (**f**) in water. Experimental points are shown as *circles*, and the curves corresponding to the best-fitting equilibria models are given as *solid lines*. The standard deviations in the e.m.f. are: 3.36 (KK), 2.41 (KAK), 2.56 (KAK2), 4.16 (KAK3), 1.47 (KAK4), and 4.8 mV (KAK5)

The titration curves for the KK, KAK, KAK2, KAK3, KAK4, and KAK5 peptides in water are shown in Fig. 2a–f. Because the experimental points closely follow the titration curves calculated with the use of the fitted acidity constants, it can be concluded that the model of two stepwise dissociation equilibria fits the experimental data very well for the four peptides: KK, KAK, KAK2, KAK4 and still rather well for KAK3 and KAK5.

The calculated p*K*_a_ values are collected in Table 1. No correction was applied for activity coefficients because first, given the value of the ionic strength (ranging from 0.002 to 0.003 mol·L^−1^; see Sect. 2.2), the corrections would amount to only 0.05 for p*K*_a_, which is below the SD of p*K*_a_ (Table 1) and, second, it is not clear to what extent the simple treatment of ions as spherical particles within the framework of the Debye–Hückel theory applies to the flexible and definitely non-spherical peptide molecules. For all peptides, the SD of the p*K*_a_s are reasonably small, which enables us to discuss them quantitatively.

**Table 1:** The two p*K*_a_ values and their ∆p*K*_a_ difference for each peptide: KK, KAK, KAK2, KAK3, KAK4, KAK5, in water, at 298.1 K obtained by fitting of the equilibrium model of dissociation to the potentiometric titration curves

	KK	KAK	KAK2	KAK3	KAK4	KAK5
p*K*_a1_	5.95 (0.12)^a^	8.73 (0.23)	9.36 (0.09)	8.20 (0.12)	9.67 (0.10)	9.93 (0.14)
p*K*_a2_	8.66 (0.13)	8.91 (0.14)	9.93 (0.17)	11.41 (0.29)	11.92 (0.27)	10.99 (0.19)
Δp*K*_a_^b^	2.71	0.18	0.57	3.41	2.25	1.06

^a^Numbers in parentheses are the standard deviations

^b^p*K*_a2_−p*K*_a1_

For all studied peptides, the p*K*_a1_ value is lower then that of the reference compound (10.5 for *n*-butylamine modeling the isolated lysine group [17]), while the second one is either smaller than 10.5 (for shorter chains) or higher (for longer chains). The value of p*K*_a1_ generally increases with the alanine chain length, which is consistent with a decreased influence of the positive electrostatic field of the other charged group at the opposite end of the chain (the distance between the two charged groups is expected to increase with chain length). For the KK peptide, the charged lysine groups are very close to each other and p*K*_a1_ (6.4) is lower by 4 units with respect to that of the reference compound. It can be observed that p*K*_a1_ does not vary very much from KAK to KAK2 and decreases to 8.20 for KAK3. Because the p*K*_a1_ values for KAK, KAK2, and KAK3 are similar, a charged group experiences a comparable electrostatic field from its counterpart in all these three compounds. Therefore, the two charged lysine groups seem to be at a comparable distance from each other in the three compounds, which, in turn, suggests that a chain reversal occurs for KAK2 and KAK3. This result agrees with the conclusion from our earlier work [13] that, for peptide chains of appropriate length that are flanked by a charged group at each end, a chain reversal might be induced with a non-polar peptide core inside to shield them from the solvent, while the charged groups outside expose them to the solvent. The difference between the p*K*_a_ values in the KAK3, KAK4, and KAK5 series decreases but, for KAK5, still does not reach the value of log_10_ 4 ≈ 0.60 characteristic of bifunctional acids with non-interacting functional groups (e.g., the dicarboxylic acids with a large methylene-spacer length) [12]. This fact suggests that the two groups interact with each other and are consequently close to each other, implying a bent shape for this peptide.

It can also be seen that there is a break in the trend in p*K*_a1_ variation in going from KAK2 to KAK3; p*K*_a1_ increases monotonically from KK to KAK2 and then decreases for KAK3. This behavior can be explained in terms of the shielding of the non-polar core by the charged lysine head groups. Until KAK3, the oligoalanine part of the chain is not large enough to form a hydrophobic core that could be shielded from the solvent. Consequently, p*K*_a1_ increases with increasing alanine spacer size from 5.95 for KK to 9.36 for KAK2 (Table 1), reflecting the increased end-to-end distance. However, if a chain reversal occurs for KAK3, and if the lysine end groups shield the alanine core from the solvent, then they are close to each other, which is reflected in the decreased value of p*K*_a1_ (8.20 for KAK3 compared to 9.36 for KAK2, see Table 1). With further increase of the alanine spacer size, the average distance between the lysine head groups becomes greater because of the increased loop entropy and, thereby, flexibility of the chain, and p*K*_a1_ increases from 8.20 for KAK3 to 9.93 for KAK5 (Table 1).

The fact that starting from KAK3, the value p*K*_a2_ is higher than that of the reference compound suggests that, after the first proton is released, the second charged group becomes partially shielded from the solvent by the non-polar oligoalanine core. The formation of hydrogen bonds with the deprotonated amino group of the other lysine residue might also contribute to the increase of p*K*_a2_, because a hydrogen-bonded proton is more difficult to dissociate.

## Conclusions

Our previous study [13] of a series of alanine-based peptides consisting of Ac–KK–(A)7–KK–NH2 (KAK), Ac–OO–(A)7–DD–NH2 (OAD), and Ac–KK–(A)7–EE–NH2 (KAE), suggested that their conformation depends on the size of the charged side chains at the ends and that the bent shape of the alanine sequence is formed by screening of this nonpolar heptaalanine core from the solvent by sufficiently short, charged side chains, provided that the length of the spacer between the charged groups is large enough to form a non-polar core and small enough to overcome the unfavorable ring-closing entropy. The results obtained in the current potentiometric-titration study, in which we used a series of alanine-based peptides with sequence Ac–K–(A)_*n*_–K–NHMe (KAK_*n*_, *n* = 0, 1, 2, …, 5), demonstrated that the p*K*_a1_ values are remarkably smaller than those for *n*-butylamine, which can be considered as a model of the isolated lysine side chain until *n* = 3. But, p*K*_a2_−p*K*_a1_ is still greater than the statistical factor of log_10_ (4) (found to be the limiting value for the difference in p*K*_a_ values for dicarboxylic acids with large methylene spacers [12]) even for KAK5. Leaving out the KK and KAK peptides from the series, for which two lysine groups are close to each other because of chain connectivity, these results suggest that the two charged groups are close to each other for KAK2 and KAK3, which implies a chain reversal for these peptides. This supports the hypothesis stated in our earlier work [13]. To obtain further evidence, conformational studies of the KAK*n* peptides are required; these are currently being carried out in our laboratory by CD and NMR spectroscopy.
